# Physician Perspectives on the Impact of Artificial Intelligence on the Therapeutic Relationship in Mental Health Care: Qualitative Study

**DOI:** 10.2196/81970

**Published:** 2025-12-30

**Authors:** Isabel B Weir, Austin M Stroud, Jeremiah J Stout, Barbara A Barry, Arjun P Athreya, William V Bobo, Richard R Sharp

**Affiliations:** 1 Biomedical Ethics Program, Mayo Clinic, 200 First Street SW, Rochester, MN, 55905, United States, 1 507-538-6502; 2 Radcliffe Humanities, Faculty of Philosophy, University of Oxford, Oxford, United Kingdom; 3 Alix School of Medicine, Mayo Clinic, Rochester, MN, United States; 4 Department of Psychiatry and Behavioral Sciences, University of Washington, Seattle, WA, United States; 5 Robert D. and Patricia E. Kern Center for the Science of Health Care Delivery, Mayo Clinic, Rochester, MN, United States; 6 Department of Molecular Pharmacology and Experimental Therapeutics, Mayo Clinic, Rochester, MN, United States; 7 Department of Psychiatry and Psychology, Mayo Clinic, Rochester, MN, United States; 8 Department of Behavioral Sciences & Social Medicine, College of Medicine, Florida State University, FL, United States

**Keywords:** artificial intelligence, AI, mental health, therapeutic relationship, patient-physician relationship, communication, interviews, family medicine, psychiatry, qualitative, providers, physicians

## Abstract

**Background:**

The therapeutic relationship is a professional partnership between clinicians and patients that supports open communication and clinical decision-making. This relationship is critical to the delivery of effective mental health care. The integration of artificial intelligence (AI) into mental health care has the potential to support accessibility and personalized care; however, little is known about how AI might affect the dynamics of the therapeutic relationship.

**Objective:**

This study aimed to ascertain how physicians anticipate AI tools will impact the therapeutic relationship in mental health care.

**Methods:**

We conducted 42 in-depth interviews with psychiatrists and family medicine practitioners to investigate physician perceptions regarding the impact of AI on mental health care.

**Results:**

Physicians identified several disruptions from AI use, noting that these tools could impact the dyad of the patient-physician relationship in ways that are both positive and negative. The main themes that emerged included potential disruptions to the therapeutic relationship, shifts in shared decision-making dynamics, and the importance of transparent AI use. Participants suggested that AI tools could create efficiencies that allow for relationship building as well as help avoid issues with miscommunication during psychotherapeutic interactions. However, they also expressed concerns that AI tools might not adequately capture aspects of the therapeutic relationship, such as empathy, that are vital to mental health care. Physicians also raised issues related to the impact that AI tools will have on maintaining relationships with patients.

**Conclusions:**

As AI applications become increasingly integrated into mental health care, it is crucial to assess how this integration may support or disrupt the therapeutic relationship. Physician acceptance of emerging AI tools may be highly dependent on how well the human elements of mental health care are preserved.

## Introduction

The therapeutic relationship encompasses the goals, tasks, and connection of the patient-physician dyad and has been cited by patients as integral to what defines high-quality mental health care and medical care, more broadly [1-3]. In discussing the therapeutic relationship, we refer to the overarching interpersonal dynamics between physicians and patients. This is inclusive of (although not limited to) the *therapeutic alliance*, a concept emphasized in mental health contexts that characterizes the collaborative partnership focused on achieving treatment goals [2]. In this paper, we use the term therapeutic relationship with the understanding that it encompasses the therapeutic alliance. In mental health care, the therapeutic relationship is characterized by empathy, cooperation, and support and is associated with increased medication compliance and positive outcomes [3,4]. Valued by both patients and clinicians, the therapeutic relationship is central to some professional codes of conduct [1]. It is the professional gold standard for patient-physician interaction, with documented advantages over other models in the establishment of patient trust in care providers, care satisfaction, treatment buy-in, and adherence [5,6].

The integration of artificial intelligence (AI) into the care of mental health conditions has the potential to expand the therapeutic relationship, but with ethically complex implications [7-10]. While advances in digital health technology are predicted to increase the quality of care and strengthen personalized psychiatry [11,12], AI advances may disrupt patient-physician interactions that have been conventionally viewed as building blocks of a therapeutic relationship [13]. For instance, AI-enabled chatbots aimed at providing behavioral support can potentially expand access to care but also create potential risks related to therapeutic misconception and inauthentic therapeutic relationships [14-16]. Similar concerns have been identified in patient-centered investigations, suggesting preferences for human involvement in therapy-based interactions [17] and a critical role for authenticity in health care contexts [18]. Furthermore, access to AI-supported clinical recommendations—by both physicians and patients—can influence communication with the potential to support and limit shared decision-making [19]. As AI tools are increasingly being developed and studied in the context of psychiatric care [20], their role in clinical assessment and emotional counseling could potentially impact the dynamics of patient-physician interactions [7]. For all their conveniences, AI tools may disrupt relationships between physicians and patients [21].

Several studies have sought to explore clinician perspectives regarding the use of AI and digital technology in mental health care. These studies report that many clinicians view empathetic care as a uniquely human skill that digital technologies are incapable of replicating [22-25]. In a therapeutic context, many clinicians believe that these technologies cannot engage in a personal relationship with patients and that removal of this relationship would omit the most effective aspect of mental health care [23,25]. Clinicians also note the importance of behavioral cues and nonverbal communication when assessing patients [24-26]. The use of AI tools may decrease patient trust, posing a barrier to relationship development and high-quality patient assessment [24]. However, others acknowledge that AI tools might facilitate better communication with patients who have not experienced adequate rapport with providers in the past [13,24]. While this previous work has charted many benefits and concerns associated with the adoption of AI tools for mental health care, direct investigation of provider perspectives on the anticipated impact that AI tools will have on the dynamics of therapeutic relationships is limited.

We reported findings on physician perspectives regarding the impact of AI tools on the patient-physician relationship, including its emotional impacts, influences on shared decision-making, and changes to communication. Our findings highlight physician perspectives on the ethical introduction of AI into health care, including key features physicians identify as relevant to supporting authentic therapeutic relationships in mental health care.

## Methods

### Overview

This paper draws upon an interview dataset that has been described previously [27]. Our prior studies focused on the perceived benefits and risks of AI tools in psychiatric medicine, identifying key factors that influence physician acceptance of these technologies. In this paper, we examined a different set of topics, focusing on the impact of these AI tools on the therapeutic relationship. In the following sections, we provide a summary of study methods. Readers may wish to consult our previous paper for additional methodological details [27].

### Recruitment

Physicians in family medicine and psychiatry specialties with experience treating major depressive disorder were invited to participate in our study via email invitations. These physicians were recruited from a single academic health system in the United States. Family medicine practitioners were included in addition to psychiatrists due to an expanded role for providing mental health care observed in the specialty [28,29].

### Data Collection

In-depth interviews exploring the adoption of AI into mental health care were conducted via Zoom (Zoom Communications). Interviews leveraged a case-based design to prompt participant discussion and had an average duration of 37 minutes. Three study team members participated in interviews (Susan H Curtis, AMS, and JJS), with 2 members present per interview.

Interviews began with general questions that explored participants’ perceptions of AI. To make the discussion more concrete, 2 to 3 hypothetical case scenarios were presented to participants. These cases highlighted different uses of AI based on tools currently marketed or under research. Cases illustrated the following AI uses: (1) a physician-facing tool that assisted a physician in prescribing medications based on patient pharmacogenomic information, (2) a patient-facing chatbot that provided cognitive behavioral therapy, (3) a physician-facing tool for differential diagnosis and associated disease risk scores, and (4) a physician-facing tool that identified patients at risk for suicide based on population health characteristics. Time limitations prevented interviewers from covering all cases in every interview. Interviewers selected cases to ensure that each was represented across the set of interviews. After presenting a case to the participant, the primary interviewer asked the participant to describe their general reactions to the presented AI device and then used structured and unstructured prompts to inquire about more specific issues of AI application. Some of these issues pertained to disclosure of AI device use, AI’s effect on the patient-physician relationship, AI’s performance relative to physicians, and whether AI impacted physician examination of patients. For instance, interviewers asked questions such as “How do you foresee this tool affecting the physician-patient relationship?” and “Do you have any concerns regarding the interactions between patients and chatbots?” The secondary interviewer took notes and asked probing questions as needed.

All interviews were audio recorded and transcribed by a professional transcription service. Transcripts were deidentified by deleting identifying nouns and role descriptions. Transcripts were reviewed by the study team to identify any errors by comparing them with the original audio recordings.

### Data Analysis

Transcripts were qualitatively coded by at least two members of the study team following an inductive approach [27]. This approach was informed by grounded theory [30], although it was agnostic to the goal of theory development. While grounded theory traditionally aims to generate an explanatory theory, our modified approach aimed to leverage the systematic coding procedures central to grounded theory to structure our analysis and identify themes. One primary coder (IBW) and 1 of 2 secondary coders (SHC and AMS) each independently coded transcripts using NVivo (Lumivero, LLC). Coders subsequently met to discuss their coding decisions, checking for intercoder consistency in codebook application and to resolve any discrepancies [31]. The codebook was revised throughout the coding process, with iterative refinement of coding definitions as insights emerged from data analysis [32]

### Ethical Considerations

The study was approved by the Mayo Clinic Institutional Review Board (protocol 21‐006191). All participants provided oral consent in accordance with institutional review board guidance. Study data presented in this manuscript have had all personally identifying details omitted to protect participant privacy. Participants enrolled on a strictly voluntary basis and did not receive compensation for their involvement.

## Results

### Overview

A total of 143 physicians were invited to participate in our study. Forty-two physicians, including 21 psychiatrists and 21 family medicine practitioners, enrolled and completed an interview. Several major themes arose concerning the dynamics of the therapeutic relationship, influences on shared decision-making, and transparency of AI tools.

### Physicians Felt That Health Care AI Could Disrupt the Therapeutic Relationship

Participants identified several ways that AI tools might impact personal interactions and disrupt the therapeutic relationship. One area of disruption stemmed from the potential of AI tools to replace interactions that would typically be handled by a clinical provider. Some physicians were particularly wary of AI tools for the purposes of psychotherapy and the potential to displace established relationships with human practitioners or limit their development. In terms of mental health care delivery, some physicians noted that they “don’t think it [AI] will replace the patient-physician relationship” (interview 35, family medicine); however, there was some concern that patients who defer to AI tools might not receive adequate care depending on the severity of their conditions or health circumstances:

*One potential downside, I don’t think it necessarily would be, but [it] could be that [the] patient likes the chatbot a lot more than going to see the human being psychologist, and if that relationship with the treating clinician dries up or isn’t there, what happens when the patient, if the patient has a spike in anxiety or becomes depressed, or has some change for the worse in clinical condition that would have otherwise been either identified by the psychologist, or the treatment plan would be adjusted if the patient were still engaged with the psychologist?*

[Interview 17, psychiatry]

In addition, physicians expressed that the experience of developing and cultivating a social relationship with the physician is supportive for patients. They noted the specific value of this relationship in psychotherapeutic contexts where conversation with a human provider may be preferable:

*I think if you start relying on like, “Oh, well, the computer algorithm said I should pick this,” it takes away some of the humanity. That is why people are benefiting from a psychiatrist, [it] is because you feel heard, and you feel like that person understands you. If it’s just popped out, I’m not sure that would be there*.

[Interview 28, psychiatry]

Physicians also highlighted the importance of open communication and the reception of emotions, such as empathy and compassion, as aspects of the therapeutic relationship. They noted the importance of being able to respond to nonverbal cues and physically sit with a person who is in pain. “There’s something about knowing your patients very well [...] being on a relationship level with them that I think is very hard to replace” (interview 04, family medicine). Physicians noted that often their patients confide in them because of this carefully cultivated therapeutic relationship and viewed it as essential to health care delivery. Some physicians viewed AI tools as limited in their capacity to capture these emotional aspects:

*I think personal connection is invaluable. [...] Again, eye contact, “I hear you,” that emotional sense there, which AI can’t necessarily do. There probably is some script that says, “I can tell this is difficult for you to do,” or just empathy-type thing[s], but just you know that it’s not a person*.

[Interview 35, family medicine]

Physicians also felt that the introduction of AI tools might create positive changes that could be supportive of the therapeutic relationship overall. For instance, they noted the potential of AI tools to streamline clinical tasks that could support increased time for patient interaction. “If it [AI tool] helped [...] facilitate the differential diagnosis, and really gave me more time to maybe create a relationship with the person” (interview 20, psychiatry). In addition, physicians saw ways using AI could be perceived as augmenting physician capabilities and would showcase to patients the effort being put into their treatment. Under this view, physicians saw AI tools as supplementing rather than replacing their role in health care delivery:

*I would hope that it would improve the relationship because it’s kind of like giving them, like with the chatbot, more tools to help [...] I would think it shows that you are building on knowledge that you have based on what’s going in the field and applying it to their treatment which I think it’s always a good thing*.

[Interview 40, psychiatry]

Physicians also noted that patient interactions with AI tools might help to avoid miscommunication and countertransference that can occur during a conversation between the physician and patient. “There’s a lot of transference, counter transference going on between the patients and the therapist. In this [AI chatbot] format, I see that as a non-issue” (interview 12, psychiatry). They expressed that adopting AI tools might facilitate better management of these issues in therapeutic contexts compared to conventional patient-physician interactions.

### Physicians Predicted That AI Would Impact Shared Decision-Making

Participants anticipated that the adoption of AI tools would influence the dynamics of shared decision-making. Physicians conceptualized interactions with AI as an extension of their clinical tasks, adding evidence-based validation to their decisions, and even potentially enhancing the patient-physician relationship by providing additional support. “I think it [AI tool] could be used to open up conversations on things” (interview 20, psychiatry). Participants anticipated that they would continue to value their own clinical judgment in shared decision-making conversations and suggested that AI recommendations would need to receive a physician’s “stamp of approval”:

*It might be different if I'm totally cut out of the process, but I'm still the—as the clinician, I'm still the critical processing node. This AI information is coming to me, and then I'm making the decision based on this additional information*.

[Interview 06, family medicine]

However, some participants felt that AI might begin to limit physician judgment by determining when physicians needed to be involved. Other physicians worried about the reductionist potential of health care AI being at odds with the aims of medicine. “The art of medicine is not—I think, incorporated in AI. The confounder that both the doctor and the patient represent” (Interview 13, psychiatry). Participants expressed concerns about being replaced as AI begins to guide decision-making, repositioning physicians in an ancillary role. “It [AI clinical decision support tool] seems like it’s infringing on my years of training and stuff like that, and experience. It’s like, well, no. I disagree with that” (Interview 16, family medicine).

Physicians felt that AI might also increase patient engagement in the shared decision-making process. Participants expected to respect patient choices but had concerns about patient interpretations of AI outputs and favoring them over a physician’s clinical expertise. For example, some participants compared patient interactions with AI to patients’ trust in “Dr. Google” (Interview 23, family medicine) despite the limitations of patients’ medical knowledge. Generally, participants were willing to attempt to broaden a patient’s preconceptions of their diagnosis and the best treatment plan through discussion:

*It'd be interesting if there’s a disagreement like the patient says, “I agree with the machine and not you.” That would be interesting. I think that'd be a good discussion to have. It’s probably not unusual that patients will come in with certain ideas on things*.

[Interview 20, psychiatry]

However, some participants anticipated that patients might be overly deferential to AI-supported physician recommendations. For some mental health patients, the physician’s office might already be an uncomfortable place that they have prepared for. They may enter with fixed opinions based on their own research. Patients may also experience cognitive entrenchment, causing them to default to information provided by AI tools and pull back from shared decision-making:

*It’s a real interesting thing 'cause in the right setting people accept what machines tell them. They come in convinced they have things that they may not, but they've got the control to access it and decide what to do there. Coming into the doctor’s office, they don't have the control, and my instinct is they'd still want human intervention*.

[Interview 18, psychiatry]

Physicians presented an idealized way to situate AI within shared decision-making. They noted that the primary interaction is between the physician and the patient, who together should decide whether to incorporate AI tools and recommendations into clinical discussions:

*It’s a partnership. It’s another tool. That’s why you say, “Okay. Yeah.” That’s how I would treat it. It’s kind of like we talked about if the computer’s the third person in the room, the third thing. It’s me and the patient and we bring the computer*.

[Interview 15, family medicine]

As an example, physicians expressed that in educating patients, they could incorporate AI recommendations while explaining their clinical judgment. In addition, physicians could encourage patients to express their reactions to treatment options and express their preferences based on the information provided by AI tools, along with physicians’ clinical recommendations:

*I use that as a tool to educate my patient. Explain to them what AI is, saying, you know, “You have this disease, we have this wonderful technology that does this kind of super-fast calculation or database search, in preparation for recommendations, and this is what it’s recommending.” Then starting the conversation, “I agree with this recommendation,” or, “I disagree and this is why”—yeah, I would use it*.

[Interview 23, family medicine]

### Physicians Felt Transparency Around AI Usage Was Key to the Therapeutic Relationship

Physicians expressed the importance of disclosing the use of AI tools with patients, highlighting the role of transparency in cultivating patient trust as a key part of the therapeutic relationship. “I think the important ingredient there is transparency between the patient and the provider and an agreed-upon way that this [AI tool] is going to be used” (interview 37, psychiatry). Physicians expected that patients would trust their discretion regarding how to incorporate AI clinical decision support recommendations, along with when to shift away from such recommendations. “Again, just like any other tool that we use, I don’t think it’s going to be unusual for them if they trust us to take care of them and to use the tools that we have” (interview 42, family medicine). Physicians felt that this trust was largely established through open communication with patients and respect for patient agreement in the use of tools and their outputs:

*Yeah, I think we have to be open and honest with our patients in everything that we do [...] I don’t think you’d have any reason to hide that information from them. Just tell them exactly what you’re doing, and that you’re using this tool to confirm what your suspicions are*.

[Interview 08, family medicine]

Physicians also noted they would determine when and how to disclose their use of AI tools in clinical decision-making based on the depth of their prior established relationship with their patients. In addition to physician-facing AI tools, participants acknowledged that making patients aware of AI-supported decision-making or expanding interactions to patient-facing AI might be daunting for patients struggling with certain mental health conditions. Participants noted that the rapport-building process allows physicians to determine whether disclosing and recommending the use of AI is appropriate and helpful in mental health care:

*Maybe even in the process of going through this with a patient, there may be a good approach, like, “When’s the right time to suggest this, once we have an established rapport and relationship?” and, “I think you’re making gains, and I think this would help you more.” I find maybe that might be where it would be helpful*.

[Interview 35, family medicine]

Physicians anticipated that they would tailor their disclosure of AI use based on the psychiatric conditions of their patients. For instance, some participants stated that they might be less inclined to use the term “artificial intelligence” with patients who have a history of paranoia. They stated that knowledge of a patient’s medical history would be useful to gauge the framing of AI disclosure and to guide the extent to which physicians explain the technical aspects of AI to patients (ie, presentation of AI as a digital tool vs an intelligent algorithm) to avoid any potential confusion. “Artificial intelligence, I think, is just one of those words [...] that might just cause some confusion*”* (interview 04, family medicine).

Some physicians highlighted the importance of disclosure to mitigate potential discomfort that could be experienced by patients. “I think, if I was a patient, I may feel a little taken aback by that [AI tool] but as long as we’re disclosing, I think, I’d be comfortable incorporating it” (interview 07, family medicine). Despite differing views on how to disclose AI tools, physicians still saw disclosure as important to cultivating patient trust. Furthermore, participants saw transparency and trust as a strong predictor of patient acceptance of AI.

## Discussion

### Principal Findings

There has been a great deal of excitement as well as skepticism concerning the adoption of AI tools in mental health care. Our study aimed to characterize physician perspectives on the impact of health care AI on the patient-physician relationship. Some of our study findings corroborate the broader literature, which examines how AI or digital technology might alter patient-physician interactions within the constraints of the therapeutic dynamic [22-26,33]. Furthermore, our results inform an emerging literature on the potential for a “digital therapeutic alliance,” which refers to the potential for a collaborative relationship between a patient and a digital mental health intervention [34]. While our results highlight physician skepticism regarding the capacity of AI tools to support authentic therapeutic relationships, they suggest several ways in which traditional therapeutic relationships might be supported by these tools. In addition, our findings point to several key considerations for physicians and patients as they choose to integrate AI into mental health care.

Physicians place a great deal of value on the therapeutic relationship and are sensitive to certain disruptions that AI tools may cause. The therapeutic relationship has been previously viewed in the face of digital technology or AI advances, with rapport building as a critical element of mental health care [26]. For instance, clinicians often give patients the freedom to choose engagement in specific rapport-building activities, promoting more personalized care and greater insight into factors that affect patient mental health concerns [26]. Such perspectives align with studies in which clinicians agreed that expression of empathy is a uniquely human skill [23]. An immediate barrier for therapy-based AI tools may be limitations on their ability to display emotional intelligence and moral capacity that are comparable to human-based care and insufficient for robust therapeutic relationships [35,36]. As a result, patients who value emotional connection may be particularly vulnerable to illusory representations of these features in AI tools and might find AI-based interactions limiting to their mental health care [15,21]. In addition, cognitive behavioral therapy–based digital tools may narrow the diversity of therapeutic activities, which can act as platforms for relationship building [26].

Moreover, physicians in our study observed empathy and emotional support as fundamental in data collection for diagnosis and treatment as well as integral to therapeutic relationships. Arguably, human skills are necessary for maximizing the type and quality of data collected [25]. Emotional openness may increase a patient’s expression of nonverbal behavioral cues that can be registered and analyzed by the clinician. As already seen in research on patient interviews using electronic medical record templates, some technology-directed interactions lack the emotional subtext of a natural conversation and may be the cause of missing data points that characterize patient symptomology [37].

Our results suggest that applications of AI in mental health care might be best received when they serve as a supplement rather than a replacement for physician input. Promotion of physician oversight over AI supports previous views that digital technology should not replace face-to-face care, that patients may perceive noncollaborative digital care as inferior, and that patients are more likely to accept clinician-driven care that places digital technology as an adjunct [25]. Participants noted several scenarios where AI tools could be helpful, such as freeing up time that can be re-allocated to patient interactions and supplementing clinical judgment. In this augmentative capacity, AI tools may enhance rather than undermine aspects of the therapeutic relationship.

Empathetic communication and transparency about therapeutic goals between physicians and patients were also a key feature in deciding to integrate AI tools. Strong rapport and mutual trust between physicians and patients not only promote more targeted patient care but also provide a platform for physician disclosure of AI use that can be tailored to patient mental health conditions. Our results suggest that, particularly in mental health contexts, there may be some additional consideration for disclosing the use of AI tools to patients. Physicians felt this was a decision best left to their discretion based on a gauge of their relationships with patients. However, lack of transparency in AI systems can cause physicians to inadequately disclose AI tools to their patients [38], which risks disrupting physician-to-patient communication.

Finally, intentional positioning of AI tools within patient-physician relationships may be necessary to minimize negative influences on shared decision-making. In prior work, clinicians have expressed that patient opinions may indeed support the best treatment plan and that patients value the freedom to endorse, question, or dispute the use of AI tools and subsequent treatment recommendations [25,39]. Physicians should be prepared to respectfully broaden patients’ perspectives of their diagnosis, given potential AI-supported clinical recommendations and interactions. In addition, physicians and patients might collaboratively address how AI tools could be positioned in care decisions such that neither party feels undercut by the role played by these technologies. Ultimately, AI is a tool that, similar to other types of medical tests, is subject to some anticipated level of error. However, such errors may be especially difficult to trace when using AI tools. Input data may undergo multiple hidden transformations before an output is generated. Given this potential and the perspectives shared by our study participants, AI-based clinical tools are best viewed as companions to clinical judgment, and not principal drivers of care decisions.

### Limitations

Our study has several limitations. First, participants were presented with different diagnostic and treatment-related AI devices as clinical cases; participants did not have the opportunity to experience using these technologies in clinical practice. Engagement with actual AI devices may have influenced participant perspectives and helped participants to more tangibly conceptualize how AI might affect the therapeutic dynamic. Second, we did not investigate how AI would improve or worsen the therapeutic relationship in a broader range of care settings beyond mental health care. Our study focused primarily on the opinions of physicians working at an academic medical center, which does not capture the full range of settings for mental health care delivery. Third, we did not collect demographic information from participants, which limits our ability to draw inferences based on participant characteristics. Finally, our study focused only on physicians and their perspectives on the therapeutic relationship. As a result, our findings represent only one side of a 2-person dynamic.

### Future Directions

Future research should look to gather perspectives from a broader array of stakeholders, including therapists, nurses, social workers, and patients, while grounding opinions in intervention-based studies. Investigation of physician perspectives on how physical AI devices impact the therapeutic relationship might better guide when and how certain AI devices should be integrated. Interventions could range from AI-driven differential diagnostic technologies inputted into electronic health record systems to behavioral health chatbots that can be downloaded on patients’ mobile devices. These studies could have a focus on access in disadvantaged regions, weighing how psychiatrists and family medicine practitioners aided by AI might be able to serve behavioral health without significant compromise to the therapeutic relationship. Additional research might focus on establishing best practices for clinicians, developers, and AI adopters that support the responsible use of AI tools. For instance, Delphi studies might be used to define optimal strategies for integrating AI tools and managing conflicts in clinical assessment. Similarly, user experience research might support the refinement of AI tool interfaces.

Our results suggest that future studies should examine patient and mental health professional views concerning the impact of AI tools on the therapeutic relationship. To fully understand this impact, a broader range of mental health professionals and other stakeholders should be included in these studies. Longitudinal engagement with these stakeholders will be critical to the successful integration of AI tools into mental health care.

### Conclusions

Without adequate consideration of the impact that AI will have on aspects of the therapeutic relationship, physicians may face unintended consequences from the adoption of AI tools and a disruption to relationships with patients. Our study supports a more comprehensive understanding of these issues by detailing some of the ways physicians anticipate AI will shift dynamics in the therapeutic relationship. As ongoing research and evaluation of AI tools in mental health care aim to support responsible clinical uses of these technologies, it is crucial that stakeholders assess their potential impact on the therapeutic relationship.

## Abbreviations

AI: artificial intelligence
